# PRKAG2 syndrome, a rare hypertrophic cardiomyopathy: a Brazilian long-term follow-up with extracardiac disorders

**DOI:** 10.31744/einstein_journal/2024AO0549

**Published:** 2024-07-10

**Authors:** Lenises de Paula van der Steld, Mario de Seixas Rocha, Ana Marice Teixeira Ladeia, Humberto Lago Livramento, Gervásio Batista Campos, Francisco Carlos da Costa Darrieux, Oscar Campuzano, Ramon Brugada

**Affiliations:** 1 Escola Bahiana de Medicina e Saúde Pública Salvador BA Brazil Escola Bahiana de Medicina e Saúde Pública, Salvador, BA, Brazil.; 2 Universidade Federal da Bahia Salvador BA Brazil Universidade Federal da Bahia, Salvador, BA, Brazil.; 3 Instituto do Coração Faculdade de Medicina Universidade de São Paulo São Paulo SP Brazil Instituto do Coração (InCor), Faculdade de Medicina, Universidade de São Paulo, São Paulo, SP, Brazil.; 4 Medical Science Department School of Medicine University of Girona Girona Spain Medical Science Department, School of Medicine, University of Girona, Girona, Spain.; 5 Cardiovascular Genetics Center University of Girona Girona Spain Cardiovascular Genetics Center, University of Girona-IDIBGI, Girona, Spain.; 6 Centro de Investigación Biomédica en Red-Enfermedades Cardiovasculares Madrid Spain Centro de Investigación Biomédica en Red-Enfermedades Cardiovasculares, Madrid, Spain.; 7 Hospital Josep Trueta University of Girona Girona Spain Cardiology Service, Hospital Josep Trueta, University of Girona, Girona, Spain.

**Keywords:** Mutation hypertrophy, Left ventricle, Wolff-Parkinson-White syndrome, Death, Sudden cardiac, Activated protein kinases/genetics

## Abstract

**Objective:**

This study aimed to provide a long-term follow-up of PRKAG2 syndrome and describe the new phenotypic aspects of the condition. PRKAG2 syndrome is a rare autosomal-dominant glycogen storage disease characterized by cardiac hypertrophy, ventricular pre-excitation, and conduction system disease. Fatal arrhythmias occur frequently.

**Methods:**

A family cohort of 66 participants was recruited. Clinical and genetic analyses were performed.

**Results:**

Median age of 36.97±17.28 years, with 69.9% being men. Nineteen subjects carried the deleterious variant p.K290I of the *PRKAG2* gene. This group experienced many malignant events, including eight pacemaker implants, three sudden cardiac deaths, five aborted cardiac arrests, four strokes, four premature neonatal deaths, two spontaneous abortions, five forceps deliveries, and 12 cesarean procedures. Extracardiac involvement, such as in neurocognitive and psychiatric disorders, has been observed only in carriers of mutations. Palpitations, Syncope, atrial fibrillation, atrial flutter, sinus pauses, and bradycardia were strongly and significantly associated with major or severe adverse events (sudden cardiac death, aborted cardiac arrest, pacemaker use, stroke, and congestive heart failure). Early diagnosis and intervention through antiarrhythmic drugs, anticoagulation, pacemaker implantation, radiofrequency catheter ablation, and cesarean section surgery improved the symptoms and survival rates. Mutations carriers were advised to avoid pregnancy.

**Conclusion:**

This study identified that the p.K291I_PRKAG2 mutation is associated with poor prognosis, highlighting the need for early intervention. Further research may uncover the potential connections between intellectual disability, miscarriage, and neonatal death in individuals with this syndrome.

## In Brief

van der Steld et al. reported that the rare variant p.K291I in the *PRKAG2* gene is linked with poor prognosis, requiring early intervention. There may be connections among intellectual disability, psychiatric disorders, miscarriages, and neonatal death in individuals with this syndrome, which should be investigated further.

## Highlights

■ PRKAG2 syndrome is a rare autosomal dominant glycogen storage disease characterized by cardiac hypertrophy, ventricular pre-excitation, and conduction system disease.

■ The rare variant p.K291I in PRKAG2 is associated with poor prognosis, highlighting the need for early intervention.

■ Early diagnosis and intervention using antiarrhythmic drugs, anticoagulants, pacemaker implantation, radiofrequency catheter ablation, and cesarean surgery may improve symptoms and survival rates.

■ The potential connections among intellectual disability, miscarriages, and neonatal deaths in individuals with this syndrome should be further investigated.

## Graphical abstract

**Figure:**
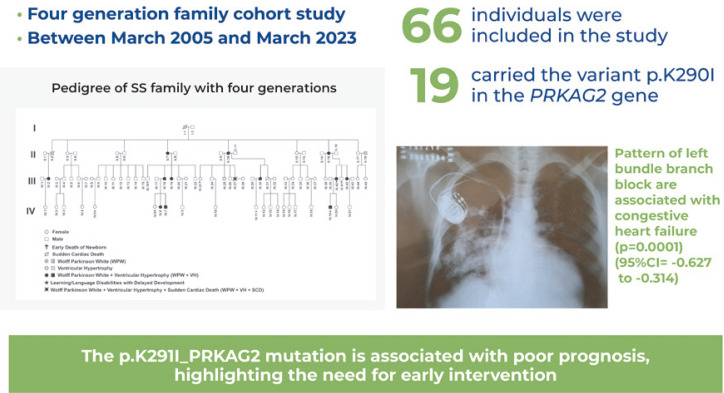

## INTRODUCTION

PRKAG2 syndrome (PS) is a rare autosomal-dominant glycogen storage disease (GSD).^(1,2)^ It affects patients in their late adolescence and is characterized by pre-excitation on an electrocardiogram (ECG). Patients experience frequent paroxysms of supra-ventricular tachycardia (SVT) and often develop conduction system disease (CSD) that may require pacemaker (PM) implantation in their fourth to fifth decade of life.^(3)^ A significant proportion of these patients experience mild-to-severe non-sarcomeric ventricular hypertrophy (VH), which can progress to dilated cardiomyopathy. Congestive heart failure (CHF), arrhythmia, and sudden cardiac death (SCD) are common.^(4,5)^ The syndrome was mapped to the locus 7q36 by MacRae et al.^(6)^ and Gollob et al.^(1)^ the gene responsible for the regulatory subunit (PRKAG2) of the 5’ AMP-activated protein kinase (AMPK).

There is a gap in the literature regarding the occurrence of neurological, psychiatric, and obstetric disorders such as mental retardation, forceps delivery, miscarriages, and premature death in individuals diagnosed with PS.

## OBJECTIVE

This study aimed to provide a long-term follow-up of PRKAG2 syndrome and describe new phenotypic aspects of this condition.

## METHODS

This longitudinal retrospective family cohort study was conducted at a hospital in Brazil between March 2005 and March 2023. Of the 89 patients, 66 were included in this study. The remaining 23 patients were excluded for reasons such as lack of interest, unavailability, or having tested positive for Chagas’ disease.

This study was conducted in accordance with the guidelines outlined in the Declaration of Helsinki. The authors were granted unrestricted access to all data and take full responsibility for their accuracy and reliability. This study was approved by the Institutional Board of the *Escola Bahiana de Medicina* (CAAE: 06512812.1.0000.5544; #165.803). The authors declare no conflict of interest. Written informed consent was obtained from all the participants and their legal guardians for those aged <18 years.

### Clinical evaluation and follow-up

Clinical data, including electronic hospital records, medical histories, and physical examinations, were collected over a period of 18 years. All participants were tested for Chagas’ disease using hemagglutination and immunofluorescence assays.

### Electrocardiographic analysis

This study examined standard 12-lead electrocardiographic recordings obtained at baseline and follow-up. To identify left ventricular hypertrophy (LVH) on ECG, the Sokolov-Lyon index criteria was used, which is described as SV1 + RV5 (or RV6) ≥35mm. In simpler terms, the sum of the amplitude of the S wave in lead V1 (SV1) and the amplitude of the R wave in lead V5 (RV5) or lead V6 (RV6) should be ≥35mm for LVH to be considered present.

The diagnosis of ventricular pre-excitation was based on the presence of a delta wave, short PR interval (≤120ms), and a widened QRS complex (≥110ms). This conduction can have a right bundle branch block (RBBB) or a left bundle branch block (LBBB) morphology. If pre-excitation was accompanied by supraventricular arrhythmia, it was classified as Wolff-Parkinson-White Syndrome. Other arrhythmias included atrial flutter (AFL), atrial fibrillation (AF), atrial tachycardia (AT), and ventricular tachycardia (VT). Conduction system disease (CSD) manifests as a bradyarrhythmia caused by sinus bradycardia, atrioventricular blocks, or sinus atrial blocks.

### Echocardiogram

All participants underwent a 2-D echocardiogram at least once a year. In case of unexplained hypertrophy in any myocardial segment, left ventricular (LV) thickness of ≥15mm septal/posterior wall ratio of 1.5 in hypertensive patients was diagnosed as LVH. Left ventricular ejection fraction (LVEF) of less than 50% was considered abnormal.

### Electrophysiological and radiofrequency catheter ablation

Electrophysiological and radiofrequency catheter ablation (RCA) was performed in 9 patients (II.17, II.10, II.15, III.2, III.19. III.29, III.30, III.42, and IV.8). All mutation carriers exhibited multiple atrioventricular accessory pathways (AP). Despite using irrigated catheters, some septal APs remain untreated because of septal hypertrophy. In eight cases, post-ablation ECGs still showed a short PQ interval, predominant delta wave (particularly in leads II, III, and aVF), and LBBB- or RBBB-type QRS configuration, indicating the presence of residual atrioventricular pathways. In two cases, RCA of 1:1 and 2:1 AFL were also observed after AP treatment, and electrophysiology (EPS) confirmed the presence of untreated anterograde APs, including the atrioventricular, nodoventricular, or fasciculoventricular pathways.

### Autopsy and microscopy

The autopsy of a 22-year-old male patient (III.27) was performed by two forensic pathologists who conducted independent examinations. No other causes of death, such as hypertension, were identified. The examination included measurement of the weight of the entire heart and the thickness of the ventricular walls. The hearts were systematically examined to identify possible regional and diffuse alterations. Samples of the septum and full-thickness myocardial sections from ventricles and auricles were fixed in formalin and embedded in paraffin. A ten-micron-thick parallel series was obtained with a microtome, mounted on slides, and stained with hematoxylin and eosin (H&E), Masson’s trichrome stain, and Periodic Acid-Schiff (PAS). For electron microscopy observation, the samples were fixed with 2.5% glutaraldehyde and paraformaldehyde (1% in 0.1 M cacodylate buffer) at pH 7.4. Samples were then washed and dehydrated in ethanol, dried, and sputtered with a 22.5nm gold layer (1.5KV) and deposition current (30mA for 2 minutes).

### Genetic analysis

In this study, genetic testing involved the collection of saliva and blood samples from all family members. Genomic DNA was extracted using a commercial Oregon kit, and the *PRKAG2 gene’s* exons and exon-intron boundaries were amplified using Veriti PCR. Purified PCR products were sequenced bidirectionally using a Big Dye Terminator v3.1 and 3130XL Genetic Analyzer. Posterior SeqScape Software v2.5 compared the results with those of the hg19 reference sequence. Rare variants were compared using the Human Gene Mutation and Genome Aggregation Database (gnomAD). Non-common genetic variants (MAF <1%) were categorized according to current ACMG recommendations.

### Groups

Subjects with pathogenic *PRKAG2 gene* variant were categorized as either affected by clinical symptoms (AI) or non-affected without clinical symptoms (NA). Major adverse cardiac events include PM implantation, aborted cardiac arrest (ACA), sudden cardiac death (SCD), hospitalization for heart failure (HF), and events leading to thrombosis or stroke.

### Statistical analysis

Statistical analyses were performed using SPSS (version 14.0; SPSS Inc., Chicago, IL, for statistical). Data were presented as mean±SD (range) or frequency (%). It was employed χ^2^, Pearson’s, Fisher’s exact, or χ^2^ tests for categorical variable relationships and two-sample Student’s *t*-tests for continuous variable group comparisons. The primary outcome was SCD, with combined events including SCD, ACA, PM, Stroke, and CHF. Kaplan-Meier curves were used to estimate the cumulative incidence, and the log-rank (Mantel-Cox) method was used to compare factors. The results were considered statistically significant at p<0.05.

## RESULTS

### Clinical features

**Figure 1:**
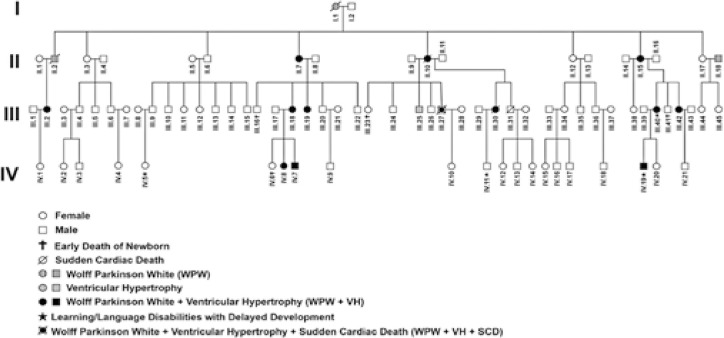
Pedigree of SS family with four generations

Of the 66 patients recruited for the study, 19 (28.8%) were categorized as affected by the disease (AI) and carried the missense variant (c.869A>T, p.K290I) in PRKAG2. The remaining 47 individuals (71.21%) were categorized as non-affected (NA). Figure 1 presents the pedigree of the four generations within this family cohort, highlighting the presence of the disease with autosomal dominant transmission. The median age was 36.97±17.28 years, with an interquartile range (IQR) of 0.3 to 73 years, and (46-69.9%) of the participants were male. All the participants were of non-Caucasian ethnicity and tested negative for Chagas’ disease. Hypertension was observed in (12-63%) of the AI Group and (4.0-10%) of the NA Group. The heart rate of the AI Group was lower than that of the NA Group (57.8±18.8) *versus* (69.6±3.2) p<0.001). Four generations of SS family pedigree.

### Natural history and clinical events

All clinical, ECG, and CHO variables were normal in the NA Group. In the AI Group, the initial signs in infancy included palpitations, syncope, seizures associated with SVT, sinus bradycardia, and pauses. Among the adult AI Group members, dyspnea, chest pain, and dizziness were reported with no muscular involvement, and creatinine kinase levels were normal.

Table 1 shows that when comparing the AI and NA Groups, there were no significant differences in age, sex, VT presence, left atrium size, and IVS/PW thickness by echocardiography. However, all other variables showed significant differences (p=0.0001). Three patients with WPW (I.1, II.2, and II.18) experienced SCD before the start of the study. Hypertension (63%), diabetes (68%), and smoking (22%) were the most common comorbidities in the AI Group.

**Table 1:** Comparison study between affected and non-affected Individuals

	AI Group n=19	NA Group n=43	p value
Age: years	36.4±16.45	36.9±17.2	0.403
Sex (Male)	13.0 (68)	17.0 (40)	0.015
Smoke	4.0 (22)	1.0 (2.4)	0.025
Palpitation	17.0 (90)	N	<0.001
Dyspnea	17.0 (68)	1.0 (2.4)	<0.001
Dizziness	13.0 (68)	1.0 (2.4)	<0.001
Myalgia	2.0 (11)	N	<0.001
Chest pain	14.0 (74)	1.0 (2.4)	<0.001
Pre-syncope	14.0 (74)	N	<0.001
Syncope	15.0 (83)	N	<0.001
Hypertension	12.0 (63)	4.0 (10)	<0.001
*Diabetes mellitus*	6.0 (68)	N	<0.001
PR interval	87.8±13.7	149.9±26.9	<0.001
QRS complexes	129.2±37.2	76.9±13.9	<0.001
QTc	414.5±41.8	335.8±42.7	<0.001
Right bundle brunch block	10.0 (60)	7.0 (37)	<0.001
Left bundle brunch block	7.0 (40)	N	<0.001
Bradycardia	14.0 (74)	4.0 (10)	<0.001
Pauses	9.0 (47)	N	<0.001
Atrial fibrillation	8.0 (44)	N	<0.001
Atrial tachycardia	6.0 (33)	N	<0.001
Atrial Flutter	8.0 (44)	N	<0.001
Supraventricular tachycardia	14.0 (78)	N	<0.001
Ventricular tachycardia	1.0 (1.7)	N	0.30
Total AV block	4.0 (21)	N	<0.001
Left atrium	39.7±9.7	30.25±4.1	0.333
Posterior wall	14.8±4.9	8.5±1.4	0.148
Interventricular septum	15.9±5.3	8.6±1.4	0.143
Left ventricular ejection fraction	57.8±18.8	69.6±3.2	0.011

Values are n (%), median (interquartile range)±SD.

AI: affected individuals; NA: non-affected individuals; QTc: average corrected QT interval measurements; AV: atrioventricular.

### Electrocardiographic findings

During the 18-year follow-up, it was found that the most common electrocardiographic features of PS in this cohort were a short PQ interval (19-100%), a delta wave, and RBBB pattern morphology (positive QRS component in lead V1, suggestive of a left-sided atrioventricular accessory pathway (AP) (10-52.63%), and LBBB pattern (6-31.58%) or right-sided patterns. These findings are consistent with those reported in the literature.^(4)^Table 1 provides descriptions of the two groups.

**Figure 2:**
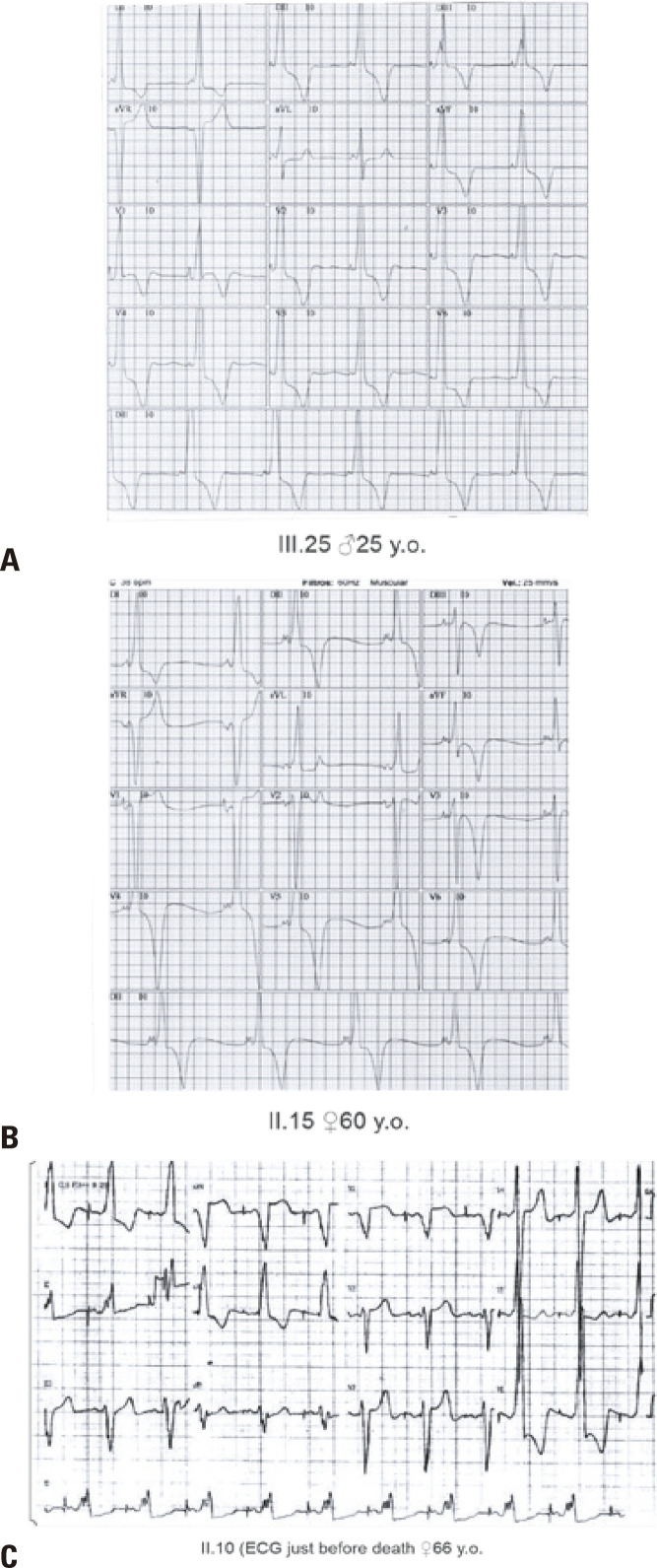
Electrocardiogram typical of PS from patients III.25, II,15, and II.10 with short PR interval, delta wave, and high voltage indicating left ventricular hypertrophy in the QRS and T wave inversion. Electrocardiogram of II.10 was done just before her death. Patients: III.25 male 25-year-old, II.15 female 60-year-old. II.10 ECG just before death female 66-year-old

Subsequently, starting at the age of 10 years, there was a notable development of QRS complex enlargement with a high amplitude (8-42%). Additionally, AF (9-47%), AFL (8-44%), AT (6-33%), nonsustained VT (1-1.7%), and ventricular repolarization alterations, such as T-wave inversion, were observed (Figure 2).

The PR interval was significantly shorter in the AI Group (87.8±13.7) compared to the NA Group (149.9±26.5) (p=0.001). QRS complexes were wider in the AI Group (129.2±37.23) and the NA Group (76.9±13.98) (p=0.001). Furthermore, the corrected QT interval was longer in the AI Group (414.53±41.77) compared to the NA Group (335.79±42.66) (p=0.001). In the AI Group, a higher incidence of CSD manifested in early childhood as an atrioventricular block, sinoatrial block, sinus pauses, sinus bradycardia, and asystole. No patient showed signs of sustained ventricular tachycardia. The mechanisms of ACA include sudden pauses (as observed in patient IV.8) and AF with anterograde conduction through the accessory pathway, leading to asystoles.

### Echocardiographic characteristics of the affected group

The mean posterior wall (PW) thickness measured 14.8±4.76%, the intraventricular septum (IVS) thickness was 25.93±5.36%, and the left ventricular ejection fraction (LVEF) was 57.8±18.8%. The NA population showed no signs of VH throughout the follow-up period.

The affected patients predominantly exhibited an asymmetric, non-obstructive septal hypertrophic pattern, mainly affecting the high and middle regions of the IVS, with preserved systolic function after the second decade of life. In the AI Group, patients II.7, II.10, II.27, and III.30 (4%–21.05%) developed left ventricular systolic dysfunction, resulting in three deaths attributed to CHF class IV/IV (N.Y.H.A.).

None of the patients had pulmonary artery wall (PW) or interventricular septum (IVS) measurements of >30mm. Specifically, (14-73.68%) of patients fell within this range, while (4-21.05%) displayed the presence of a patent oval foramen, as outlined in table 1.

### Electrophysiologic studies and radiofrequency catheter ablation

Electrophysiology and RCA were performed in 10 patients with AI (II.7, II.10, II.15, III.2, III.30, III.30, III.33, III.41, III.43, and IV.8) due to symptomatic and spontaneous SVT. Multiple AP with Mahaim fibers were identified in all patients with AIs. The AP locations were as follows: two left laterals (LL), two right laterals, two parahissians, four right posteroseptal, four left posteroseptal, and 10 Mahaim fibers. No sustained VT was observed during the study period. Some AP remain untreated owing to septal hypertrophy, even with the use of irrigated catheters. Two patients experienced 1:1 and 2:1 AFL episodes after RCA of the APs and subsequently underwent another EPS and RCA. The patient was diagnosed with Mahaim fibers and AFL.

### Major or serious adverse events

Only AI presented the major events: (3-15.7%) sudden cardiac death (SCD), (5-26.31%) aborted cardiac arrest (ACA), (8-42.1%) pacemakers (PM) implantations, (1-5.26%) implantable cardioverter-defibrillator (ICD) with one inappropriate shock due to AF, (4-21.5%) hospitalizations due to class III or IV N.Y.H.A of CHF, (3-15.78%) embolic strokes, (3-15.78%) thrombosis, and (1- 5.25%) bacterial endocarditis.

Palpitations and syncope, were observed from the first year of life in the AI Group, leading to SVT and sudden pauses. Early implantation of a PM helped manage dizziness and syncope and supported antiarrhythmic and CHF treatments.

At age 11, Subject IV.7, a male with WPW and VH, experienced convulsive syncope episodes correlated with pauses, resulting in a dual-chamber PM. However, he continued to experience syncope with multiple episodes of SVT, which were effectively treated with RCA targeting the AP. At the age of 19, he was diagnosed with bacterial endocarditis, which resulted in ACA due to electrode displacement. This incident necessitated the replacement of the entire cardiac pacing system. The patient was scheduled for another RCA procedure because of recurrent SVT.

### Thrombo-embolism, neurocognitive and psychiatric disorders in affected individuals

Patients: IV.5 female 14-year-old and IV.19 male 26-year-old have WPW, neurocognitive delay, seizures, difficulties walking, learning disabilities, anxiety, aggressiveness, speech disorders, and changes in mood and behavior.

Patient III.25, a 28-year-old male with WPW and sinus bradycardia, had experienced syncope and seizures since childhood. At the age of 25, he underwent implantation of a dual-chamber PM, and the symptoms disappeared. Additionally, he developed inferior vena cava thrombosis, which was initially managed with warfarin and subsequently transitioned to rivaroxaban. This patient also presented with convergent strabismus; mental retardation; difficulties in initiating, planning, and organizing thoughts; below-average learning abilities; behavioral and speech disorders; reasoning challenges; and stunted growth (1.50m; weight, 48kg).

Patient II.10, a 64-year-old female with WPW and VH, developed HF, renal failure, intracardiac thrombus, and stroke at the age of 58 years after sustaining AF and 2:1 AFL. She underwent RCA of the AFL and an AP, which was located inside the coronary sinus, under anticoagulation with warfarin, followed by apixaban. Unfortunately, her health deteriorated, and she ultimately died of sepsis and heart failure.

Patient III.30, a 41-year-old female with WPW, VH, underwent RCA for multiple AP (right lateral, right posteroseptal, and Mahaim). She had a medical history of pulmonary embolism and thrombosis in her right leg, both linked to atrial fibrillation (AF) and AFL when she was 36 years old. The patient was initially administered dabigatran. However, as her condition progressed, leading to renal failure and severe CHF, categorized as New York Heart Association (NYHA) class IV, the therapy was shifted to warfarin. Regrettably, her health continued to deteriorate, and she ultimately succumbed to ailments.

Patient II.7**,** a 66-year-old female with WPW, VH, and ICD, had a transient embolic stroke at the age of 60 years and was treated with rivaroxaban 20mg/day.

### Spontaneous abortions, forceps delivery, and newborn premature deaths in AI

Spontaneous abortions (2-10.52%), premature newborn deaths (4-21.05%), (5-27.7%), (12-63.1%), and premature newborn deaths (4-21.05%) were observed only in the AI Group. Five women reported preeclampsia.

### Risk factor association and Kaplan-Meier curve

**Figure 3:**
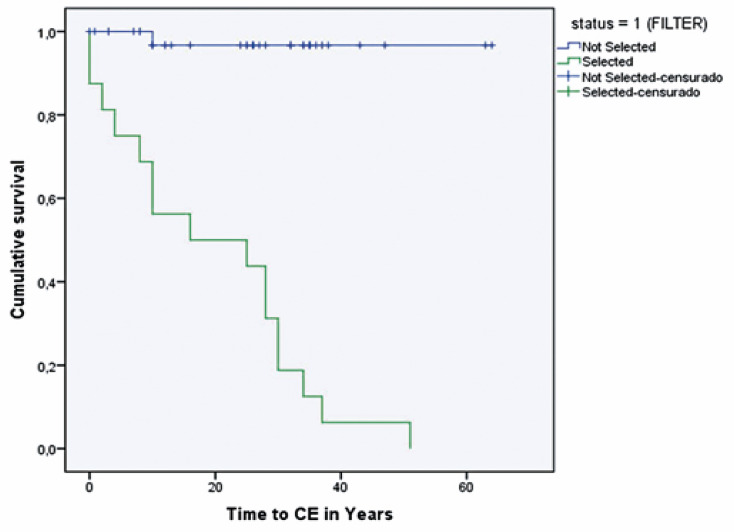
Kaplan-Meier curve shows the cumulative survival probabilities for major events. Non-affected has a blue curve and is affected by the PRKAG2 variant in green

Figure 3 illustrates the Kaplan-Meier curve representing the combined occurrence of major cardiovascular events, including SCD, ACA, PM implantation, and stroke. The curves for individuals with and without the condition revealed the profound impact of the disease on survival and its long-term implications, which have been evident since childhood.

Patients with this condition have a poor prognosis and a significantly elevated risk of complications. Notably, the risk factors associated with these combined events included syncope (p=0.0001), atrial fibrillation (AF) (p=0.0001), AFL (p=0.0001), complete atrioventricular block (AVB) (p=0.001), pauses (p=0.0001), and bradycardia (p=0.0001). These factors exhibited a robust, positive, and statistically significant correlation with the occurrence of combined events (CE), including SCD, ACA, PM, stroke, and CHF. Cox regression analysis did not show statistical significance when comparing these variables.

### Electrophysiological studies and radiofrequency catheter ablation

Electrophysiological studies and RCA were performed in ten affected patients (II.7, II.10, II.15, III.2, and III.19). III.29, III.30, III.42, IV.7. IV.8). All AI Group presented multiple AP. Septal hypertrophy poses a significant challenge to the complete elimination of APs, particularly those in this region, even when an irrigated catheter is used.

After ablation, ECG results in a significant number of cases (8-42%) that continued to display a short PQ interval, a prominent delta wave particularly evident in leads II, III, and aVF, and QRS configurations reminiscent of LBBB or RBBB. These ECG findings provided clear and precise evidence of the persistence of another residual atrioventricular pathway related to the Mahaim fibers. Several years after ablation, three of these patients experienced atrial fibrillation (AF) as well as 1:1 and 2:1 AFL, further confirming the ongoing presence of anterograde accessory pathways (atrioventricular, nodoventricular, or fasciculoventricular) that had not been effectively treated. Consequently, these patients required subsequent RCA.

### Autopsy

An autopsy of a 25-year-old male (III.27) with CHF and SCD was performed.

His medical history included an episode of ACA at 16 years of age due to atrial fibrillation (AF) with anterograde conduction through the accessory pathway (AP). Subsequently, he underwent an RCA procedure for the left lateral AP. At 22 years of age, he experienced another ACA incident due to AFL with a total atrioventricular (AV) block and asystole (Figure 2). The patient received a dual-chamber PM. By the age of 24, he developed sustained 2:1 AFL and CHF, classified as NYHA Class III/IV. An RCA procedure was performed; however, the patient developed SCD at 25 years of age.

**Figure 4:**
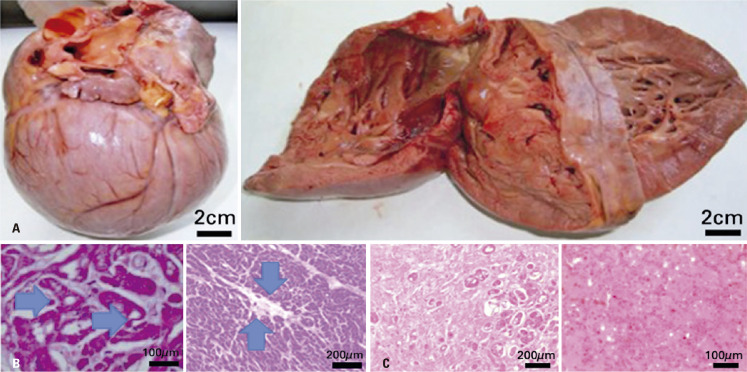
A) Macroscopic Aspect of Heart Disease: 22-year-old male WPW, LVH, CSD. Asymmetrical hypertrophy of the ventricles, with important thickness of interventricular septum. Weight: 786g; B) Microscopic examination: Extensive fibrosis permeated by atrophied and vacuolated myocytes (hypertrophied). Abundant magenta positive granules (blue arrows) in cardiomyocyte cytoplasm indicating glycogen deposition; C) Lung with congested septal vessels and diffuse intra-alveolar edema

On examination, the heart weighed 786g and displayed a smooth epicardium. The lung segment, weighing 142g, had a smooth pleura and a brownish cutting surface, as depicted in figure 4, illustrating both the macroscopic and microscopic aspects of the heart. The myocardium exhibited varying thicknesses, measuring 0.5cm and 1.8cm in the ejection cones of the pulmonary and aorta arteries, respectively. Notably, there were areas of slightly clearer and poorly defined myocardium in the middle third of the interventricular septum, the upper third of the left ventricle (LV), and the transition area between the septal IV-VD regions. The coronary arteries remained unaffected, and no abnormalities were observed in the aorta. Microscopic examination revealed VH due to glycogen storage disease, characterized by the accumulation of glycogen within the vacuoles (A). Additionally, extensive fibrosis (B) was observed within the cardiac tissue, which contributed to the development of CHF and pulmonary edema (C).

### Genetic analysis

Genetic analysis revealed a rare missense variant (c.869A>T, p.K290I) in the *PRKAG2* gene. No other rare variants were identified in any known genes associated with this phenotype. This rare variant induces a nucleotide 869 in exon 7 of the *PRKAG2* gene, Lysine (Lys-K-) changes to isoleucine (Ile-I-) at nucleotide 869 in exon 7 of the PRKAG2 gene. This rare variant is not found in global population databases, is considered deleterious in ClinVar, and is predicted to be pathogenic *in silico*. This rare variant was considered Likely Pathogenic (LP), following the current ACMG recommendations (PP3, PP5, PM1, PM2). The rare variant identified in the index case was segregated among all available family members (19 relatives). It is important to note that all clinically affected patients carried the rare variants. Pedigree analysis of this Brazilian family demonstrated autosomal dominant transmission of a trait that causes HCM and WPW.

### Study limitation

Notably, most affected patients do not have pacemakers compatible with MRI technology.

## DISCUSSION

This was an 18-year follow-up of carriers of the p.K290I_*PRKAG2* rare deleterious variant in a family cohort with a clinical profile dominated by cardiac manifestations and evidence of psychiatric, neurological, and obstetrical problems. AMPK is highly expressed in cardiac tissue, liver, pancreas, skeletal muscle, brain, and placenta.^(7,8)^

Similar to the reports of Burwinkel et al., a severe form of PS in patients with the p.Arg531 Gln mutation is characterized by early onset and death within the first 3 months of life.^(9)^

This study revealed extracardiac involvement similar to Danon’s syndrome. It includes psychiatric disturbances such as anxiety and mood disorders, neurocognitive issues such as learning disabilities, and mild cognitive deficits that require a multidisciplinary approach. Frequent spontaneous abortions, premature newborn deaths, forceps births, and cesarean deliveries have also been observed. The causality of these issues remains unknown; however, they require attention. It’s important to note that these alterations were not seen in the Control Group.^(10,11)^

Kim et al. confirmed that the correlation of glycogen deposits in mice with ventricular pre-excitation in the PS and VH, independent of GSD, was caused by enhanced insulin sensitivity and protein kinase B activation. In the present study, this was observed in patients with AI with diabetes mellitus and hypertension; however, more studies are needed to correlate causality.^(12)^

Arad et al. reported that chronotropic incompetence typically occurs in the third or fourth decades of life.^(13)^However, in this cohort, clinical onset ranged from the intrauterine period to early childhood (IV.7, IV.10, III,27). During the second decade of life, there is a noticeable decline in LV systolic function caused by the sustained presence of AF and AFL through the septal AP; this results in tachycardiomyopathy, particularly in the presence of LBBB.^(14,15)^

Similar to the findings of Sternick et al., multiple fast accessory pathways were observed, along with decremental accessory connections and/or fasciculoventricular pathways.^(16,17)^The ECG of the fasciculoventricular pathways showed varying patterns, such as an anteroseptal accessory pathway and others with a midseptal accessory pathway. We believe that the lack of treatment of Mahaim fibers after RF ablation could be attributed to significant VH.

In the absence of PM support, antiarrhythmic drugs were poorly tolerated by the patients in the AI Group. Amiodarone was the most effective drug for controlling atrial tachyarrhythmias; however, 5.26% of AI patients developed hyperthyroidism, and 10.52% developed hypothyroidism single ICD (II.7) was implanted in the study group, which triggered only one inappropriate shock due to AT. Unfortunately, this patient died due to complications arising from the extraction of excess electrodes, which caused a fracture in one of them. The selection of patients who would benefit from an implantable cardioverter-defibrillator (ICD) for primary prevention is a complex process. Implantable cardioverter-defibrillator implantation should be considered only when sustained VT is present. A recent study suggests that the decision to implant ICD in primary prevention should be made on an individual basis. The present study consider the risks of inappropriate shocks and complications. Dual Chamber pacemakers can improve the quality of life prevent asystole and support the use of antiarrhythmic drugs.^(18)^

This study found that ACA occurred due to AF with anterograde conduction through the AP (2-10.5%) and abrupt asystole (3-15.9%), consistent with previous research. Electrophysiologic studies can help determine the risk level for individuals with WPW and rapid SVT and may prevent the need for ICD implantation for primary prevention. Radiofrequency catheter ablation has a positive impact on outcomes, resulting in a reduction in symptoms such as palpitations, syncope, and presyncope, as well as a significant decrease in hospitalizations.^(19,20)^

The study revealed that an Aberrant Intraventricular Conduction Pattern of LBBB was significantly associated with CHF (p=0.0001) (95% confidence interval [95%CI]: -0.627 to -0.314). It is well documented that LBBB and large QRS complex can cause ventricular desynchronization, leading to CHF.

As stated in the literature, atrial flutter and AF are not well tolerated and are associated with thromboembolic events, rapid deterioration of left ventricular function, and increased hospitalization. Early diagnosis and intervention with antiarrhythmic drugs, anticoagulants, PM implantation, RCA, and cesarean section improved symptoms and survival. Bradycardia pauses, AF, AFL, AV block, and LV EF were associated with malignant events and should be treated early.

## CONCLUSION

The present study identified that the p.K291I_PRKAG2 mutation is associated with a poor prognosis, highlighting the need for early intervention. Further research may uncover the potential connections between intellectual disability, miscarriage, and neonatal death in individuals with this syndrome.
